# Alopecia Areata

**Published:** 1912-08

**Authors:** 


Alopecia Areata.—Its Causative Factors and Therapy.—Bechet, (*New York Med. Jour.,* June 29, 1912) believes that the disease presents two distinct varieties, the contagious and the trophoneurotic.

Errors of refraction, sudden nervous shock from fright, grief, accidents, etc., may be responsible. Stelwagon reports a case covering almost the entire scalp resulting from a blow on the head.

In France a number of epidemics have occurred and French observers consider the disease practically only from its contagious aspect.

Sabouraud considers the disease contagious and has found a microbacillus in the upper dilated portion of the diseased follicle which he calls, le microbacille de l'utricule peladique. It is impossible to distinguish it from the acne comedo and seborrheic bacillus of Hodara by any physical or biologic means.

Bechet believes in both local and general treatment. Locally he applies resorcin in 2.5—6% solutions with cantharides and capsicum twice daily with a circular motion. Pure carbolic acid applied with a small swab and vigorously rubbed over a small area at a time is useful. The same spot not to be touched again until two weeks have elapsed.

